# Incidence Trends and Risk Prediction Nomogram for Suicidal Attempts in Patients With Major Depressive Disorder

**DOI:** 10.3389/fpsyt.2021.644038

**Published:** 2021-06-23

**Authors:** Sixiang Liang, Jinhe Zhang, Qian Zhao, Amanda Wilson, Juan Huang, Yuan Liu, Xiaoning Shi, Sha Sha, Yuanyuan Wang, Ling Zhang

**Affiliations:** ^1^Beijing Key Laboratory of Mental Disorders, The National Clinical Research Center for Mental Disorders, The Advanced Innovation Center for Human Brain Protection, Beijing Anding Hospital, Capital Medical University, Beijing, China; ^2^Peking University HuiLongGuan Clinical Medical School, Beijing HuiLongGuan Hospital, Beijing, China; ^3^Department of Psychology, Faculty of Health and Life Sciences, De Montfort University, Leicester, United Kingdom

**Keywords:** prediction model, suicidal attempt, major depressive disorder, Chinese population, nomogram

## Abstract

**Background:** Major depressive disorder (MDD) is often associated with suicidal attempt (SA). Therefore, predicting the risk factors of SA would improve clinical interventions, research, and treatment for MDD patients. This study aimed to create a nomogram model which predicted correlates of SA in patients with MDD within the Chinese population.

**Method:** A cross-sectional survey among 474 patients was analyzed. All subjects met the diagnostic criteria of MDD according to the International Statistical Classification of Diseases and Related Health Problems 10th Revision (ICD-10). Multi-factor logistic regression analysis was used to explore demographic information and clinical characteristics associated with SA. A nomogram was further used to predict the risk of SA. Bootstrap re-sampling was used to internally validate the final model. Integrated Discrimination Improvement (IDI) and Akaike Information Criteria (AIC) were used to evaluate the capability of discrimination and calibration, respectively. Decision Curve Analysis (DCA) and the Receiver Operating Characteristic (ROC) curve was also used to evaluate the accuracy of the prediction model.

**Result:** Multivariable logistic regression analysis showed that being married (OR = 0.473, 95% CI: 0.240 and 0.930) and a higher level of education (OR = 0.603, 95% CI: 0.464 and 0.784) decreased the risk of the SA. The higher number of episodes of depression (OR = 1.854, 95% CI: 1.040 and 3.303) increased the risk of SA in the model. The C-index of the nomogram was 0.715, with the internal (bootstrap) validation sets was 0.703. The Hosmer–Lemeshow test yielded a *P*-value of 0.33, suggesting a good fit of the prediction nomogram in the validation set.

**Conclusion:** Our findings indicate that the demographic information and clinical characteristics of SA can be used in a nomogram to predict the risk of SA in Chinese MDD patients.

## Introduction

Suicide is widely prevalent and constitutes a clinical and public health concern. Close to eight hundred thousand people die annually by suicide every year. Furthermore, suicide is the second leading cause of death among people aged 15–29 globally. Suicide accounted for 1.4% of all deaths worldwide, making it the 18th leading cause of death in 2016. Suicide occurs all over the world, however, 79% of global suicides occur in low- and middle-income countries (1). According to Silverman et al's. research, suicidal behavior includes suicidal thoughts, suicide plans, and suicide attempts (SA) (2). Suicide and SA are widely prevalent on a global scale among psychiatric patients (3). A prior history of SA is a significant predictor of eventual death by suicide (4). In addition, a previous SA is the strongest risk factor for subsequent SA and suicide death (5, 6). MDD is the most common psychiatric disorder among people who die by suicide (7) and a well-established risk factor for SA (8). Globally, the lifetime risk of SA in people with MDD is estimated between 16 and 40% (9), showing a wide range that requires further exploration to better understand the actual risk percentage.

Worldwide, SA in people with MDD is constituted as an increasing clinical and public health concern (10, 11), with less investigation of the rate of suicide and its related risk predictors in China (12). In China the percentage of MDD with SA is reported to be between 14.3 and 25% (13). Previous studies explored the prediction of suicide by machine learning (14–16), and the risk factors of suicidal thought in adults based on decision tree analysis (17). However, these studies do not provide sufficient information on clinical implications to be implemented. In a previous study on suicidality in MDD patients, when compared with the full remission period the SA rate was 21-fold during the acute phase of remission and 4-fold during the partial remission period (18). Identifying the risk factors associated with SA in people with MDD during the acute phase of remission is vital for early identification to reduce death by suicide. Despite the substantial literature on risk and protective factors for SA, there does not exist studies that explore the prediction model of SA using socio-demographic information and clinical characteristics.

This study aimed to provide a prediction model to identify the correlates with SA and diagnosed MDD. The researchers' hypothesized that demographic information and clinical characteristics would interact to predict SA in MDD patients. The hypothesis tested the association of demographic information and clinical characteristics, both interactively and individually, with the risk of SA among MDD patients using survey data.

## Materials and Methods

### Participants

This study was cross-sectional and retrospectively analyzed using a clinical database from Beijing Anding Hospital (a tertiary hospital for psychiatric disorders in Beijing, China). All participants were recruited from December 2013 to November 2016 from the Department of Major Depressive Disorder, Beijing Anding Hospital. All participants met the diagnostic criteria of MDD according to the International Statistical Classification of Diseases and Related Health Problems 10th Revision (ICD-10) and were diagnosed by an experienced psychiatrist. The data of participants was then collected into the electronic medical record system by two experienced psychiatrists. The personal information of subjects was removed to provide a layer of anonymity to the patients. All patients had previously been informed of and agreed that the information in their medical record could be shared anonymously for the purpose of research. The study protocol was approved by the Ethics Committee of Beijing Anding Hospital.

Initially, 545 participants' data was retrieved, and 71 were excluded. The excluded participants were due to various reasons, including, incomplete sociodemographic information and/or clinical variables. The researchers reviewed the complete medical history from patients and patients with other psychiatric illnesses including schizophrenia, schizoaffective disorder, bipolar disorder, personality disorder, and intellectual disability were excluded. Also, those who had a history of a psychiatric illness that presented with a comorbidity of alcohol or drug abuse were excluded. Finally, 474 patients with complete records were included in the study. Anti-depressant medications, including anti-depressants and atypical anti-psychotics, for the enrolled patients did not affect the participants' data from being included in the study.

### Materials

The socio-demographic information and clinical variables included age, duration, number of episodes, age of onset, number of hospitalizations, the features of anxiety and psychiatric symptom, marital status, income, level of education, and employment status.

The assessment of suicide was completed by a psychiatrist and was part of the medical record. Within the medical record, lifetime suicidal thoughts, and suicide plans were defined as a “yes” response to the questions: “Have you ever thought about suicide?” and “Have you ever had a plan for how to kill yourself?”. SA was defined as a “yes” response, in the record, to the question: “Have you ever tried to kill yourself?”. Based on the responses, the MDD patient records were then classified into two groups: patients with SA (MDD-S) and patients without SA (MDD-N). Patients who had suicidal thoughts or suicide plans were enrolled in the MDD-N group, as they had not acted on the thoughts or plans. It is important to note that suicidal ideation, suicide plan, and suicide preparation were not the criteria for enrollment in the group MDD-S. The definition of SA can only be satisfied if the patient has engaged in specific suicidal attempts (such as drug overdose, etc.) at any time in the past. These definitions are commonly used by previous researches studying suicide (19–21).

### Statistical Analyses

In this study, continuous and categorical variables were, respectively, described using mean (standard deviation) and count (percent). The Chi-Squared Test, *t*-test, and the Wilcoxon Rank-Sum Test were used to assess the differences between the two groups and were based on demographic information and clinical characteristics at baseline. Logistic Regression Analyses was then used to identify suicide risk factors associated with the demographic information and clinical characteristics before and after adjusting for sex and age. Effect-size estimates are expressed as Odds Ratio (OR) and at a 95% confidence interval (CI). Meanwhile, the nomogram was created based on the independent prognostic factors determined by applying both forward and backward stepwise selection methods in the logistic regression model.

The statistical analyses of this study follows the statistical methods of previous studies (22, 23). A prediction nomogram was created using significant risk factors by assigning a graphic preliminary score to each of the predictors with a point ranging from 0 to 100. The preliminary scores were then summed to generate a total score. The prediction nomogram was lastly converted to the logit and then to an individual probability (from 0 to 100%) of the patients with SA. The performance of the nomogram was evaluated by Harrell's concordance index (C-index) and the calibration plot (24). Generally, C-index >0.7 reflects a well-fitted feature of the predictive model. Independent significant variables were used to develop the nomogram. The internal validation was performed using the bootstrap method. The Hosmer–Lemeshow test was used to assess goodness of fit of the nomograms. A Hosmer–Lemeshow test *p* > 0.05 meant that the nomograms showed good fit. A function based on the variance inflation factor was used to check for the collinearity of variables that were included in the regression equation. A variance inflation factor higher than 10 implies multi-collinearity (25).

In order to explore the accuracy of the prediction model, the researchers created the basic model using four factors: age, time of onset, employment status, and sex. The prediction accuracy gained by adding significant risk factors was assessed using both calibration and discrimination viewpoints. Integrated Discrimination Improvement (IDI) (26, 27) was used to evaluate the discrimination capability of significant risk factors. Calibration capability was calculated using the −2log likelihood ratio test. The researchers used Akaike Information Criteria (AIC) to evaluate the predicted probability of adding a significant risk factors to the actual risk and the global fit of the modified risk model (28). Furthermore, Decision Curve Analysis (DCA) was enrolled to inspect the net benefit of this addition (29). In this curve, the X-axis denotes threshold probability, and the Y-axis denotes net benefits. Moreover, the Receiver Operating Characteristic (ROC) curve was also calculated in this study.

The estimation of study power was performed using the PS-Power Simple Size software (version 3.1.2). The generated nomogram, DCA, and ROC curve were generated by R-language (version 3.5.2). Statistical analyses of this study were conducted using the STATA software special Release 14.0 (Stata Corp, TX). Results were considered statistical significance at *p* < 0.05.

## Results

### Demographic Characteristics

Four hundred and seventy-four hospitalized MDD patients were involved in the present analysis (mean age: 45.2 years old, SD = 13.6), including 290 females and 184 males. The mean duration of hospitalization for MDD patients was 7.8 years (MDD-N: 7.1 years and MDD-S: 9.8 years), and the mean age of onset of MDD was 37.5 years old for all patients (MDD-N: 38.3 years old and MDD-S: 35.1 years old). The duration of illness, number of episodes, age of onset, employment status, and level of education had significant differences between the MMD-N group and the MDD-S group. Meanwhile, there were no differences in age, sex, marital status, and income level between the MMD-N and MDD-S groups (Table 1).

**Table 1:** Demographic information and clinical characteristics.

	**MDD-N**	**MDD-S**	
	**(*n* = 347)**	**(*n* = 127)**	***P*-value**
Age (years)^*^	45.3 (13.3)	44.9 (14.5)	0.932
Sex (*n*, %)			0.361
Female	208 (59.9)	82 (64.6)	
Male	139 (40.1)	45 (35.4)	
Duration (year)^*^	7.1 (8.4)	9.8 (9.7)	0.006
Number of episodes^*^	2.6 (2.2)	2.7 (1.5)	0.011
Age of onset^*^	38.3 (12.9)	35.1 (12.7)	0.024
Number of hospitalization^*^	1.3 (1.1)	1.4 (0.8)	
Anxiety features (*n*, %)	165 (47.6)	50 (39.4)	
Psychotic symptom (*n*, %)	62 (17.9)	24 (18.9)	
Marital status (*n*, %)	285 (82.1)	100 (78.7)	0.068
Employment Status (n, %)	297 (85.6)	95 (74.8)	0.006
Income (*n*, %)			0.626
0–1,000 Yuan	30 (25.7)	12 (9.4)	
1,000–3,000 Yuan	118 (34.0)	47 (37.0)	
3,000–5,000 Yuan	91 (26.2)	34 (26.8)	
5,000–Yuan	72 (20.7)	25 (19.7)	
UN	36 (10.4)	9 (7.1)	
Level of education (*n*, %)			<0.001
Primary school	20 (5.7)	8 (6.3)	
Junior high school	70 (20.2)	48 (38.8)	
High school	119 (34.3)	44 (34.6)	
College school	138 (39.8)	27 (21.3)	

* *Mean (SD)*.

### Identification of Risk Factors

**Figure 1:**
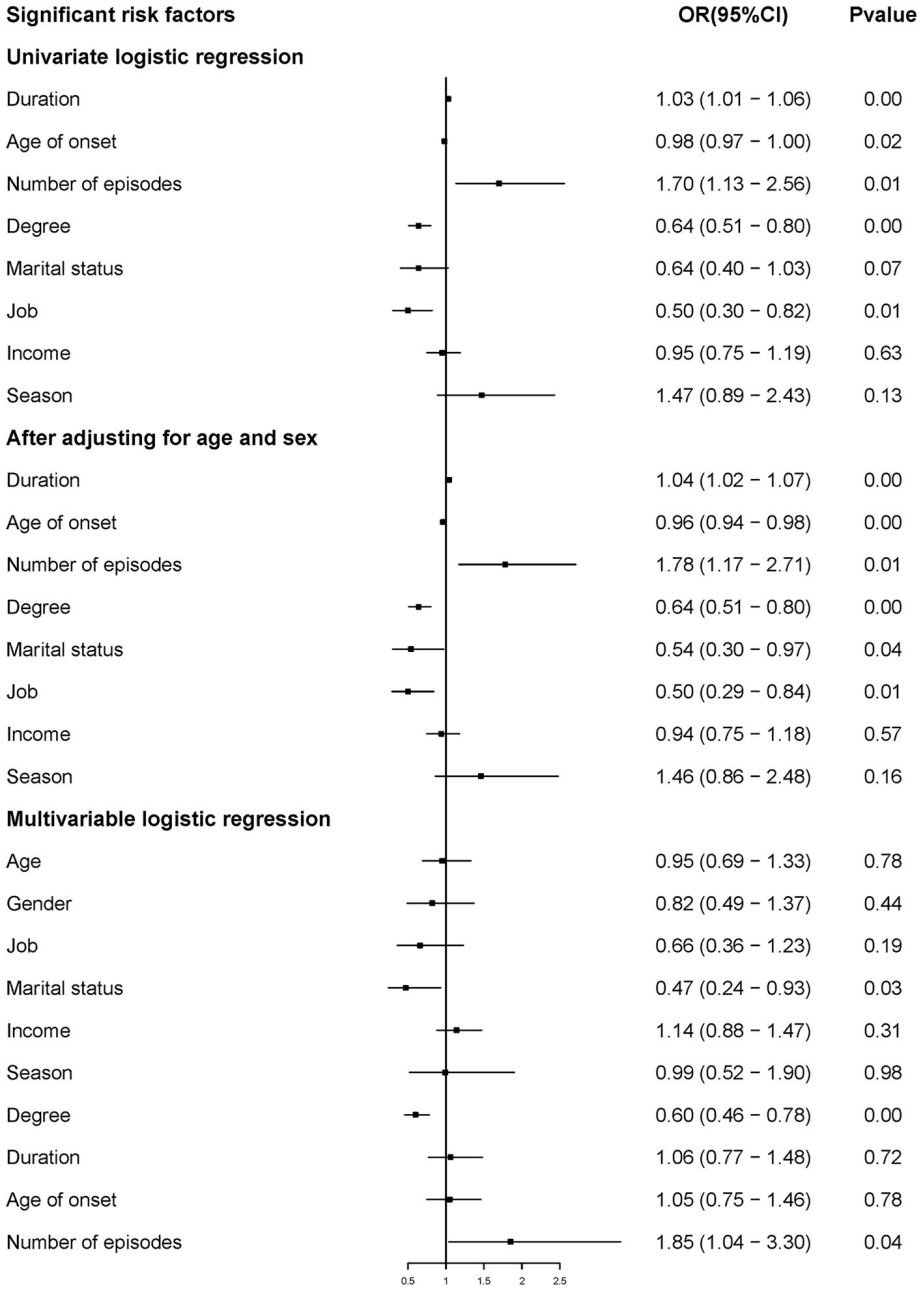
Risk prediction of demographic information and clinical characteristics for the risk of SA.

The effect-size estimates of the examined factors in correlation with demographic information and the risk factors of SA (after adjusting for sex and age) are shown in Figure 1. In this study, the risk prediction of duration of illness, age of onset, and level of education was significantly associated with SA (*p* < 0.01) after adjusting for age and sex. The risk prediction of the number of episodes, marital status, and employment status was also significantly associated with SA (*p* < 0.05). Furthermore, after multi-variable logistic regression analysis, marital status (OR = 0.473, 95% CI: 0.240 and 0.930) and level of education (OR = 0.603, 95% CI: 0.464 and 0.784) decreased the risk of the SA. However, number of episodes (OR = 1.854, 95% CI: 1.040 and 3.303) increased the risk of SA in this study.

### Prediction Model

**Figure 2:**
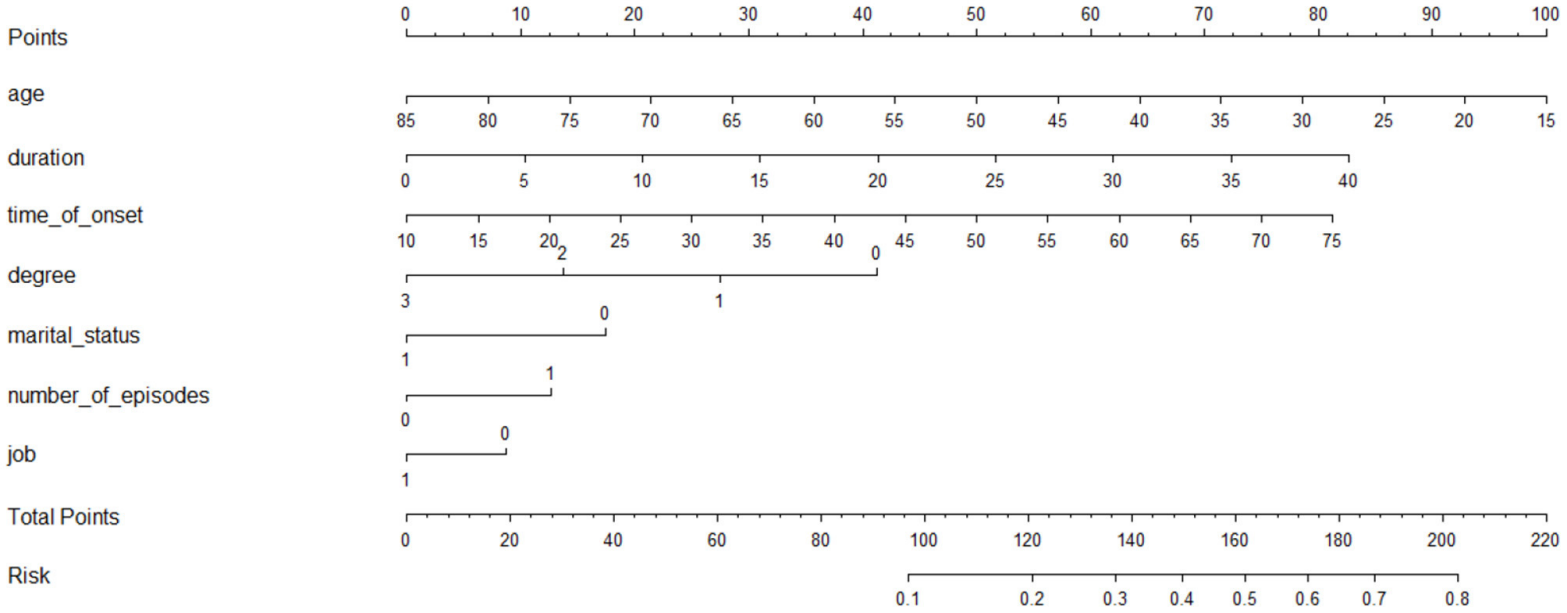
The prediction nomograms of risk factors for SA in MDD patients.

**Figure 3:**
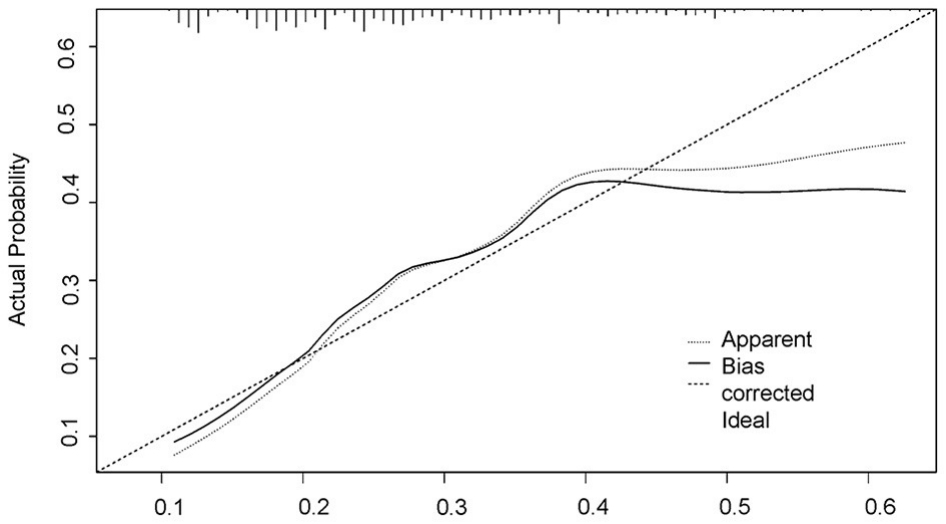
The logistic calibration curve of the prediction nomograms of risk factors for SA in MDD patients.

As in Figure 2, a nomogram model was developed to predict the risk of the SA based on the above significant factors in the logistic regression analyses, including: age, duration, age of onset, level of education, marital status, number of episodes, and employment status. In the prediction model, participants with a level of education up to 12 years were defined as 3, 2 if educated for 9–12 years, 6–9 years was defined as 1, and <6 years was defined as 0. If the subjects were married it was defined as 1, otherwise, it was 0. If number of episodes were greater than one, this was set at 1 in the nomogram. Patients who had a stable job were defined as 1, otherwise they were defined as 0. For example, in the nomogram, a MDD patient aged 50 years old would receive 50 points if they had a duration of 5 years (10 points), with an age of onset of 45 years old (43 points), a level of education up to junior high school (28 point), unmarried (18 points), number of episodes ≥1 (14 points), and who had a job (0 points) would have a total score of 163 point. The probability of SA would then approximately be estimated as 50%. The calibration curve of the nomogram demonstrated good agreement between predicted and observed risk of SA. The C-index of the nomogram was 0.715, and turned into 0.703 in the internal (bootstrap) validation sets (Figure 3). A c-index value of 0.70 or higher indicates that the nomogram had a good consistency. The Hosmer–Lemeshow test yielded the *P* = 0.33, suggesting a good fit of the prediction nomogram in the validation set. Multi-collinearity was tested using variance inflation factors (VIF), with VIF higher than 10 indicating multicollinearity (25). The variance inflation factors of the nine potential predictors ranged from 1.69 to 8.57, indicating no multi-collinearity.

### Prediction Accuracy Assessment

**Figure 4:**
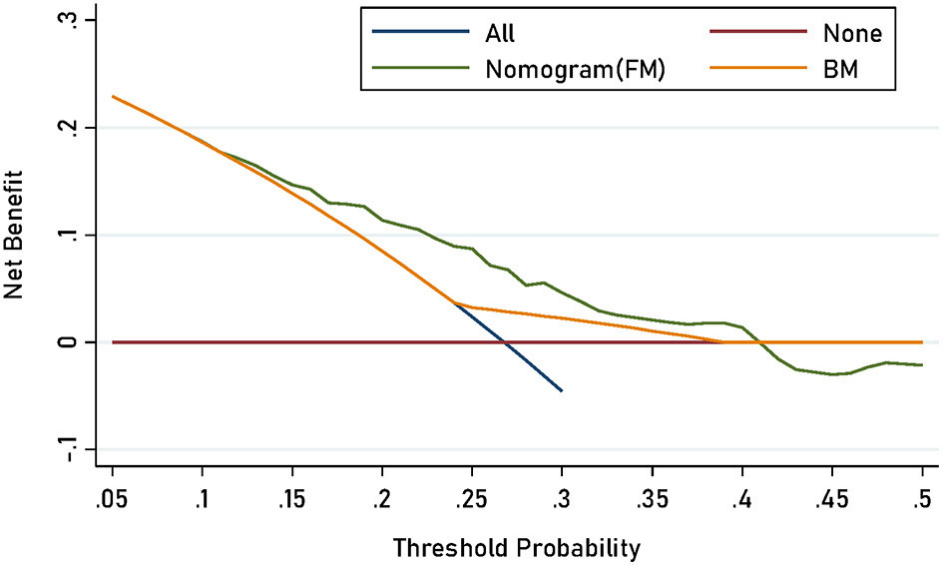
DCA for the prediction nomograms of risk factors for SA in MDD patients.

**Figure 5:**
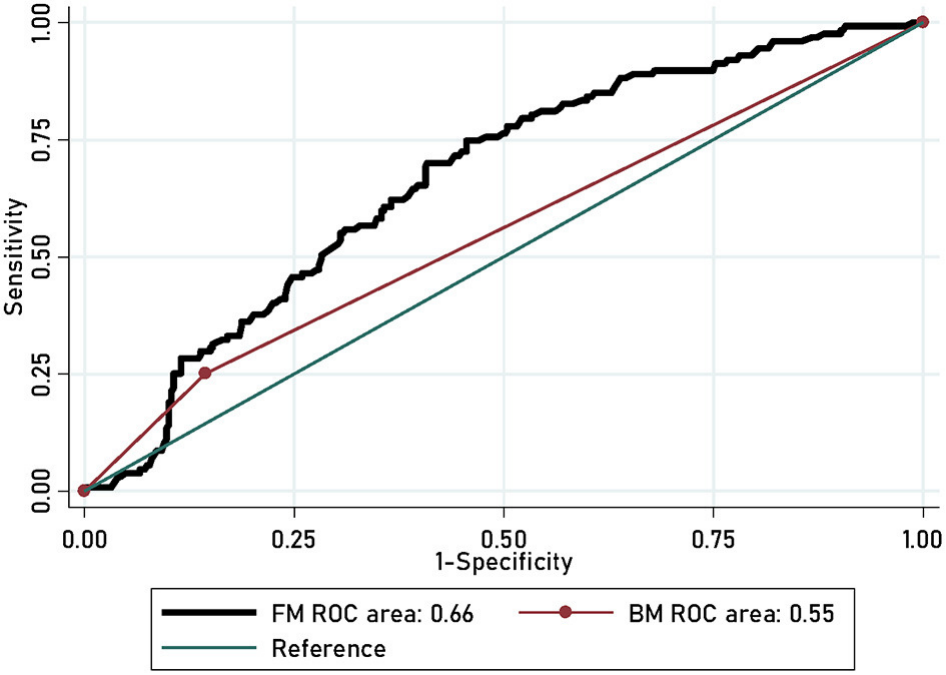
ROC plots of risk factor for SA in MDD patients.

The power to detect the incidence trends for SA was estimated to be marital status (74%), degree (17.8%), duration (4.9%), time of onset (5.0%), and number of episodes (78.8%), respectively. Table 2 shows the prediction accuracy gained by separately adding aforementioned risk factors to the basic model. Reduction in AIC statistics was >10 after adding risk factors to the basic model. A difference value >10 indicates that the model has a good calibration capability (20). Additionally, likelihood ratio tests revealed statistical significance (*p* < 0.001). From discrimination aspects, IDI indicated that the addition of risk factors to the basic model significantly improved the power of discrimination (*P* < 0.001), which was further confirmed by DCA and ROC (Figures 4, 5).

**Table 2:** Prediction accuracy gained by adding risk factors to basic model for the risk of SA.

**Statistics**	**Basic model**	**Full model**
**Calibration**		
AIC	543.9	533.8
BIC	564.8	571.3
LR	Ref.	20.33
LR (*P*)	Ref.	<0.001
**Discrimination**		
IDI	Ref.	<0.001
ROC curve	Ref.	13.01
ROC curve (*P*)	Ref.	<0.001

*AIC, Akaike information criterion; BIC, Bayesian information criterion; LR, likelihood ratio; IDI, integrated discrimination improvement; ROC, Relative operating characteristic curve; Ref., reference group*.

*Basic model included age, time of onset, job; Full model included age, time of onset, job, duration, level of education, marriage times of episode*.

## Discussion

This is the first study to explore the interaction between demographic information and clinical characteristics to predict SA in MDD inpatients. The nomogram model was created to predict the risk of SA. The full model had good prediction accuracy.

In the nomogram model, the younger age of MDD patients played a key role in predicting risk factors of SA. Borentain et al.'s findings indicated a higher proportion of suicidal ideation among young MDD patients, with almost 50% under 35 years of age (30). Similar findings were also reported in earlier research (31, 32) confirming that the younger the MDD patient, the higher the risk of SA. Our prediction model supported the findings in these above studies.

In this nomogram model the duration of having MDD is an important risk factor to predict the risk of SA. A previous study also showed that duration was a critical risk factor for suicidal behavior in MDD inpatients (33). Rice and colleagues reported that MDD patients may be exposed to a higher risk of suicidal behavior if they do not receive effective treatment at an early stage (34). The result suggested that early diagnosis and treatment for MDD patients may contribute to reducing the risk of SA.

This study showed that a late-onset of MDD was associated with an increased risk of SA when compared to early-onset MDD. A similar result was also mentioned in a previous study of 3,284 adults, which found that the factors of SA were evaluated with longer exposure time (years at risk) (10). It concluded that older patients with MDD who were exposed to related risk factors for longer periods of time had a higher risk of SA. However, other findings showed that if onset of MDD occurred before adulthood there may be a higher risk of suicidal behavior, which was inconsistent with the findings of this study (10, 35, 36). This is likely a result of cultural differences, decades of studies have found that there are cultural protective factors and risk factors that are associated with suicidal behavior (37, 38). Previous studies have also supported that the risk factors of SA vary among people in different countries and cultures (39, 40). Meanwhile, many patients are hospitalized before suicidal ideation progresses to SA. The selection of such a sample would cause the result to deviate from real world data (41).

As expected, having a status of single, having a low-level education, and having a higher number of episodes of depression, impacted on the risk of SA. Inconsistent with previous studies (42, 43), the current study also found that status of single was a significant risk factor for SA. In the nomogram model for this study, as predicted, unmarried MDD patients exhibited an increased risk of possessing SA when compared to married patients. Compared to married patients, unmarried patients may be more vulnerable to SA due to a lack of protective factors, such as social and family support, an outlet for relieving stress, etc. (44). Looking at the impact of level of education on the risk of SA, this study's finding was corroborated in line with several previous studies, suggesting that a lower level of education was associated with increased risk of SA (45, 46). Previous research demonstrated that a lower education level was predictive of SA, independent of clinical factors (47). This could be due to those with lower education having increased odds of divorce, unemployment, and falling into poverty (48), which are risk factors for SA. Meanwhile, less successful social functioning, as mentioned above, was related to a greater risk of SA (49–51). In our study, the researchers also found that the number of episodes was a predictive factor of SA in this study. A similar result was also been found in a representative study that showed that recurrent MDD appeared to confer a higher risk for suicidal behavior (52). Meanwhile, Chaudhury et al. also emphasized the importance of the higher number of episodes of depression in SAs (50). The researchers speculate that the effect of the higher number of episodes on the risk of SA could be explained by the repetition of depression during the depressive episodes, or the extended presence of deep despair.

Psychotic symptom, anxiety features, and sex showed no significant differences between the MDD and S-MDD groups. The results indicate that symptoms and sex may not be risk factors when predicting SA in MDD patients. This finding is in contrast to previous studies (53, 54). Previous research at general hospitals in China found significant risk factors for MDD in outpatients, included being female and having a comorbidity with an anxiety disorder (12). The researcher team considered the possibility that they did not find the correlation in symptoms and sex might have been impeded by enrolled patients who were hospitalized. Therefore, further research should clarify the differences between in and outpatients in a nomogram model to predict the risk of SA between outpatients and hospitalized MDD patients.

This study had some limitations. First, in terms of living situations, the study did not take into account circumstances other than being married and single (unmarried), which ignores patients who were divorced or separated. Future research should explore the living situation instead of marital status to be more inclusive. Second, because the study was retrospective there was no control over the variables collected. Future research should explore a more exhaustive set of socio-demographic variables. The retrospective study precludes further comments on the cause-effect relationship between physical examination, laboratory tests (homocysteine, serum total cholesterol, triglycerides, free thyroxine) (55, 56), and SA. All participants were hospitalized and may not reflect the general population, requiring further external validation in future studies. Finally, an insufficient sample size might have influenced the validity of the nomogram model. It was deduced that the poor corresponding power of risk factors was also related to the small sample size. Referring to previous studies, more than 1,000 subjects are the recommended sample size of a nomogram [(22), 57]. Considering the small sample size, the researchers did not divide our data into three sub-groups (train, validation, and test). However, the researchers did use bootstrap re-sampling to verify the results, appropriately.

In conclusion, our findings indicated that age, duration, age of onset, level of education, marital status, numbers of episode, and employment status may serve as early-stage predictive factors for SA. The prediction model could enhance earlier identification, effective prevention, and improve the prognosis and treatment for SA in MDD patients. The prediction model created shows good prediction accuracy when administered to Chinese patients with MDD.

## Data Availability Statement

The original contributions presented in the study are included in the article/supplementary material, further inquiries can be directed to the corresponding author/s.

## Ethics Statement

Written informed consent was obtained from the minor(s)' legal guardian/next of kin for the publication of any potentially identifiable images or data included in this article.

## Author Contributions

SL and JZ: writing-original draft and writing-review and editing. QZ: conceptualization. AW and JH: formal analysis and writing-review and editing. YL: formal analysis, methodology, and writing-review and editing. XS and SS: formal analysis. YW and LZ: data curation and writing-review and editing. All authors contributed to the article and approved the submitted version.

## Conflict of Interest

The authors declare that the research was conducted in the absence of any commercial or financial relationships that could be construed as a potential conflict of interest.
